# Serum arterial lactate concentration predicts mortality and organ dysfunction following liver resection

**DOI:** 10.1186/2047-0525-2-21

**Published:** 2013-10-07

**Authors:** Matthew G Wiggans, Tim Starkie, Golnaz Shahtahmassebi, Tom Woolley, David Birt, Paul Erasmus, Ian Anderson, Matthew J Bowles, Somaiah Aroori, David A Stell

**Affiliations:** 1 Hepatobiliary Surgery, Plymouth Hospitals NHS Trust, Derriford Hospital, Derriford Road, Plymouth, Devon PL6 8DH, UK; 2 Peninsula College of Medicine and Dentistry, University of Exeter and Plymouth University, Research Way, Plymouth, Devon PL6 8BU, UK; 3 Department of Anaesthetics, Plymouth Hospitals NHS Trust, Derriford Hospital, Derriford Road, Plymouth, Devon PL6 8DH, UK; 4 Centre for Health Statistics, Tamar Science Park, Davy Road, Plymouth, Devon PL6 8BX, UK

**Keywords:** Liver, Hepatectomy, Post-operative care

## Abstract

**Background:**

The aim of this study was to determine if the post-operative serum arterial lactate concentration is associated with mortality, length of hospital stay or complications following hepatic resection.

**Methods:**

Serum lactate concentration was recorded at the end of liver resection in a consecutive series of 488 patients over a seven-year period. Liver function, coagulation and electrolyte tests were performed post-operatively. Renal dysfunction was defined as a creatinine rise of >1.5x the pre-operative value.

**Results:**

The median lactate was 2.8 mmol/L (0.6 to 16 mmol/L) and was elevated (≥2 mmol/L) in 72% of patients. The lactate concentration was associated with peak post-operative bilirubin, prothrombin time, renal dysfunction, length of hospital stay and 90-day mortality (*P* < 0.001). The 90-day mortality in patients with a post-operative lactate ≥6 mmol/L was 28% compared to 0.7% in those with lactate ≤2 mmol/L. Pre-operative diabetes, number of segments resected, the surgeon’s assessment of liver parenchyma, blood loss and transfusion were independently associated with lactate concentration.

**Conclusions:**

Initial post-operative lactate concentration is a useful predictor of outcome following hepatic resection. Patients with normal post-operative lactate are unlikely to suffer significant hepatic or renal dysfunction and may not require intensive monitoring or critical care.

## Background

Despite advances in both operative technique and peri-operative care, liver resection is associated with post-operative mortality rates of 0% to 22% (median 3.7%) [1] and morbidity rates of 12.5% to 66% including liver dysfunction [2,3], renal dysfunction [4] and bile leak [5,6]. Factors associated with peri-operative complications and death include patient age [7,8] and gender [9,10], hospital annual number of liver resections undertaken [9,11], pathologic origin of liver tumour [9,11], pre-operative liver and renal dysfunction [8,10], diabetes [12,13], chronic liver disease [7,9], and the peripheral neutrophil to lymphocyte ratio (NLR) [14]. Operative factors associated with outcome include blood loss [8,10] and transfusion [15,16], extent of liver resection [15,17], duration of surgery [18], simultaneous extrahepatic procedures [15,19], and the use of the Pringle manoeuvre [16,20].

Therefore, many factors affect outcome after liver surgery which have not been incorporated into a single scoring system. The American Society of Anesthesiologists (ASA) grade and Portsmouth Physiologic and Operative Severity Score for the enUmeration of Mortality and morbidity (P-POSSUM) scores are used in the risk prediction of many types of surgery [21,22] including liver surgery [23]. However, these scores may not be applicable to the unique stresses of liver resection. One of the main reported causes of mortality following liver resection is post-hepatectomy liver failure (PHLF) [24]. Although the ‘50-50 criteria’ of serum bilirubin of >50 μmol/L and prothrombin index (laboratory’s calculated mean normal prothrombin time (PT) divided by the patient’s observed PT) of <50% measured on the fifth post-operative day have been shown to be associated with death due to PHLF [2], an earlier prediction system may be clinically more useful in guiding therapy. Furthermore, failure of multiple organ systems may contribute to death following liver resection and there is a need for a global peri-operative measure to predict the risk of developing significant post-operative morbidity and death.

Lactic acid is a by-product of anaerobic metabolism that is subsequently metabolised in the liver during gluconeogenesis [25]. Hyperlactataemia has been shown to be associated with increased mortality and morbidity in a critical care setting [26,27], in patients with liver failure [28], sepsis [29] and following trauma [30]. Similar relationships have been shown in the post-operative setting following pancreatic resection [31] and other major abdominal surgery [32], cardiac surgery [33] and after hepatic transplantation [34].

The primary aim of this study was to determine if the first post-operative arterial lactate concentration (‘initial lactate’) is associated with adverse outcomes following liver resection including 90-day mortality, length of hospital stay (LOS), and renal and hepatic dysfunction. The secondary aim was to determine which pre- and intra-operative risk factors are associated with initial lactate concentration following liver resection.

## Methods

This study was a retrospective analysis of a prospectively maintained database of all patients undergoing liver resection since July 2005. Routine patient characteristics, laboratory data and intra-operative details were retrieved. Pre-operative liver-directed chemotherapy was administered to selected patients following discussion at a regional multidisciplinary team meeting. A period of recovery of at least six weeks was allowed following cessation of chemotherapy before undertaking surgery. The P-POSSUM scoring system was used to calculate the physiological score [21]. Prior to resection, the operating surgeon makes a visual assessment of the condition of the liver parenchyma and records this as normal or abnormal. Liver resections were performed using standard techniques with a Cavitron Ultrasonic Surgical Aspirator™ (CUSA; Tyco Healthcare, Mansfield, MA, USA) dissector. Hepatic inflow occlusion was used in a minority of cases where there was excessive blood loss. Anaesthetic techniques include the routine use of invasive arterial blood pressure monitoring, central venous pressure monitoring (CVP) (using a target CVP of <5 cm H20) and epidural anaesthesia. Liver resections were defined according to the Brisbane classification [35] and the number of removed segments recorded. Intravenous fluid replacement was minimised during the resection phase to decrease venous pressure. After removal of the surgical specimen, a pause in surgical activity is routinely planned to allow haemostasis and intravenous volume replacement with 0.9% saline or Hartmann’s solution at the anaesthetist’s discretion. Patients are usually returned to the High Dependency Unit (HDU) after surgery with full invasive monitoring, except for minor resections in fit patients who are returned to the general ward.

The serum lactate was recorded from an arterial blood sample taken immediately prior to abdominal closure or immediately on arrival in the HDU. The arterial lactate in the normal population is below 1.6 mmol/L whereas in a critical care setting <2 mmol/L is more commonly accepted in acutely stressed patients [36].

Serum biochemistry tests and coagulation assays were performed on all patients in the first 24 hours post-operatively and the tests repeated according to clinical course. The peak measurement of bilirubin and PT were recorded and used for analysis. A PT index of <50% corresponds to a PT >24 s. Similarly peak post-operative creatinine levels were obtained and renal dysfunction was defined according to the Risk, Injury, Failure, Loss, and End-stage kidney disease (RIFLE) criteria [37]. Renal dysfunction in categorical analyses was defined as any increase in serum creatinine of ≥1.5-fold from the pre-operative baseline. The length of hospital stay was measured from day of surgery to day of discharge and was expressed as a natural logarithm. Ninety-day mortality was recorded.

The association between initial serum lactate concentration and continuous outcomes was investigated using a multiple linear regression model as well as Spearman’s rank correlation. To overcome increasing variance with the mean a natural log transformation was used. Binary variables were investigated using univariate regression. Potential associations between initial lactate concentration and pre- and intra-operative factors were tested using univariate regression or chi-square test at the level of *P* < 0.25 [38], as appropriate. Significant variables in the univariate analysis were included in the multivariate regression model and were considered to be significant if *P* < 0.05. All analyses were carried out using the statistical package R 2.1.14 [39].

Confirmation was obtained from the South West Health Research Authority that under the harmonised Guidance Approval for Research Ethics Committees (REC), REC review was not required because patient data was collected in the course of their normal hospital care and was anonymised for research purposes. No patient consent was required for this study.

## Results

In the study period 501 patients underwent liver resection for whom an initial lactate measurement was available in 488. The indications for surgery, pre-operative and operative details are shown in Table 1. Results of blood tests are shown in Table 2 and the main post-operative outcome measures are summarised in Table 3. The median number of biochemistry tests performed per patient in the first five post-operative days was 4 (0 to 6) and coagulation assays was 3 (0 to 6). It was not necessary to administer clotting factors to any surviving patients between postoperative days 1 to 5. Peak abnormalities in PT and bilirubin usually occurred early in the post-operative course and tended to improve over five days (Table 2). Post-operatively, 118 patients (24.1%) had a serum bilirubin ≥50 μmol/L. Minor abnormalities in PT were commonly noted, though only 15 patients (3.1%) developed a PT >24 s. Although a small number of patients remained jaundiced at the time of discharge, only one patient fulfilled the ‘50-50 criteria’ at day five. The median length of hospital stay was seven days (range 2 to 78) with 90% of patients having a LOS between two and 15 days. Twelve patients (2.5%) died within 30 days of surgery and 23 died within 90 days of surgery (4.7%). The most common cause of death was liver failure, which occurred in 11 of 23 patients. Four patients died from ongoing malignancy (of whom three had undergone non-curative resections) and two patients died from sepsis without evidence of liver failure. The remaining deaths were attributed to pulmonary embolus, heart failure, anastomotic leak following colonic resection, bleeding peptic ulcer, strangulated hernia and peritonitis.

**Table 1:** Pre-operative and intra-operative characteristics of 488 patients undergoing liver resection

**n = 488**			**Median (range)**	**Count (%)**
Age (years)			65 (21–90)	
Gender	Female			216 (44.3)
	Male			272 (55.7)
Pathology of resected specimen	Benign			40 (8.2)
	Primary	Hepatocellular carcinoma		30 (6.1)
		Cholangiocarcinoma		36 (7.4)
		Other		35 (7.2)
	Secondary	Colorectal metastases		291 (59.6)
		Other		56 (11.5)
Pre-operative liver-directed chemotherapy	Yes			173 (35.5)
	No			315 (64.5)
Body mass index			26 (16–54)	
P-POSSUM physiologic score			16 (12–32)	
ASA grade	1			49 (10.1)
	2			315 (64.7)
	3			121 (24.8)
	4			2 (0.4)
Pre-operative diabetes	Yes			55 (11.3)
	No			433 (88.7)
Pre-operative bilirubin (μmol/L)			9 (2–162)	
Pre-operative alkaline phosphatase (U/L)			95 (34–1190)	
Pre-operative albumin (g/L)			44 (10–53)	
Pre-operative creatinine (μmol/L)			78 (40–430)	
Pre-operative glomerular filtration rate (ml/min)	≤90			158 (33.2)
	>90			318 (66.8)
Neutrophil to lymphocyte ratio (NLR)			2.47(0.3-17.3)	
Operation number	1st			453 (92.8)
	2nd			30 (6.1)
	3rd			5 (1.0)
Surgeons assessment of liver parenchyma	Normal			314 (65.3)
	Abnormal			167 (34.7)
Surgical approach	Open			440 (90.2)
	Laparoscopic			48 (9.8)
Radiofrequency ablation (RFA) included	Yes			22 (4.5)
	No			466 (95.5)
Operation	Right hemihepatectomy			142 (29.1)
	Extended right hemihepatectomy			65 (13.3)
	Left hemihepatectomy			55 (11.3)
	Extended left hemihepatectomy			24 (4.9)
	Left lateral sectorectomy			45 (9.2)
	Wedge resection only			127 (26.0)
	Other			30 (6.1)
Wedge resection included	Yes			182 (37.3)
	No			306 (62.7)
Bile duct reconstruction included	Yes			43 (8.8)
	No			445 (91.2)
Synchronous bowel procedure	Yes			22 (4.5)
	No			466 (95.5)
Curative intent	Yes			442 (90.6)
	No			46 (9.4)
Number of segments resected			4 (1–6)	
Estimated blood loss	<100 ml			2 (0.4)
	101-500 ml			240 (49.7)
	501-1000 ml			167 (34.6)
	>1000 ml			74 (15.3)
Units of red cells transfused			0 (0–26)	

**Table 2:** Post-operative blood tests for 488 patients undergoing liver resection

**n = 488**		**POD 0**	**POD 1**	**POD 2**	**POD 3**	**POD 4**	**POD 5**
Bilirubin	Tested (%)	393 (81)	385 (79)	324 (66)	255 (52)	213 (44)	200 (41)
	Median (range)	21 (5–170)	27 (6–211)	21 (4–195)	19 (3–167)	18 (4–179)	19 (1–186)
Prothrombin time	Tested (%)	387 (79)	317 (65)	233 (48)	170 (35)	135 (28)	107 (22)
	Median (range)	16.3 (12.2-32.4)	18.0 (12–200)	18.0 (12.6-39.4)	16.1 (11.2-37.2)	15.3 (11.6-30.6)	15.4 (12.0-26.4)
Creatinine	Tested (%)	425 (87)	458 (94)	374 (77)	288 (59)	241 (49)	226 (46)
	Median (range)	70 (30–319)	70.5 (29–377)	64.5 (26–686)	60.5 (28–518)	59 (25–611)	60 (26–292)

**Table 3:** Post-operative outcomes for 488 patients undergoing liver resection

**n = 488**	**Median (range)**	**Count (%)**
Peak bilirubin (μmol/L)	29 (4–445)	
Peak prothrombin time (s)	17.6 (12.4-200)	
Length of stay (days)	7 (2–78)	
Renal dysfunction	None	450 (92.2)
	Risk (>1.5x pre-operative creatinine)	17 (3.5)
	Injury(>2x pre-operative creatinine)	12 (2.5)
	Failure (>3x pre-operative creatinine)	5 (1.0)
90-day mortality		23 (4.7)

**Figure 1:**
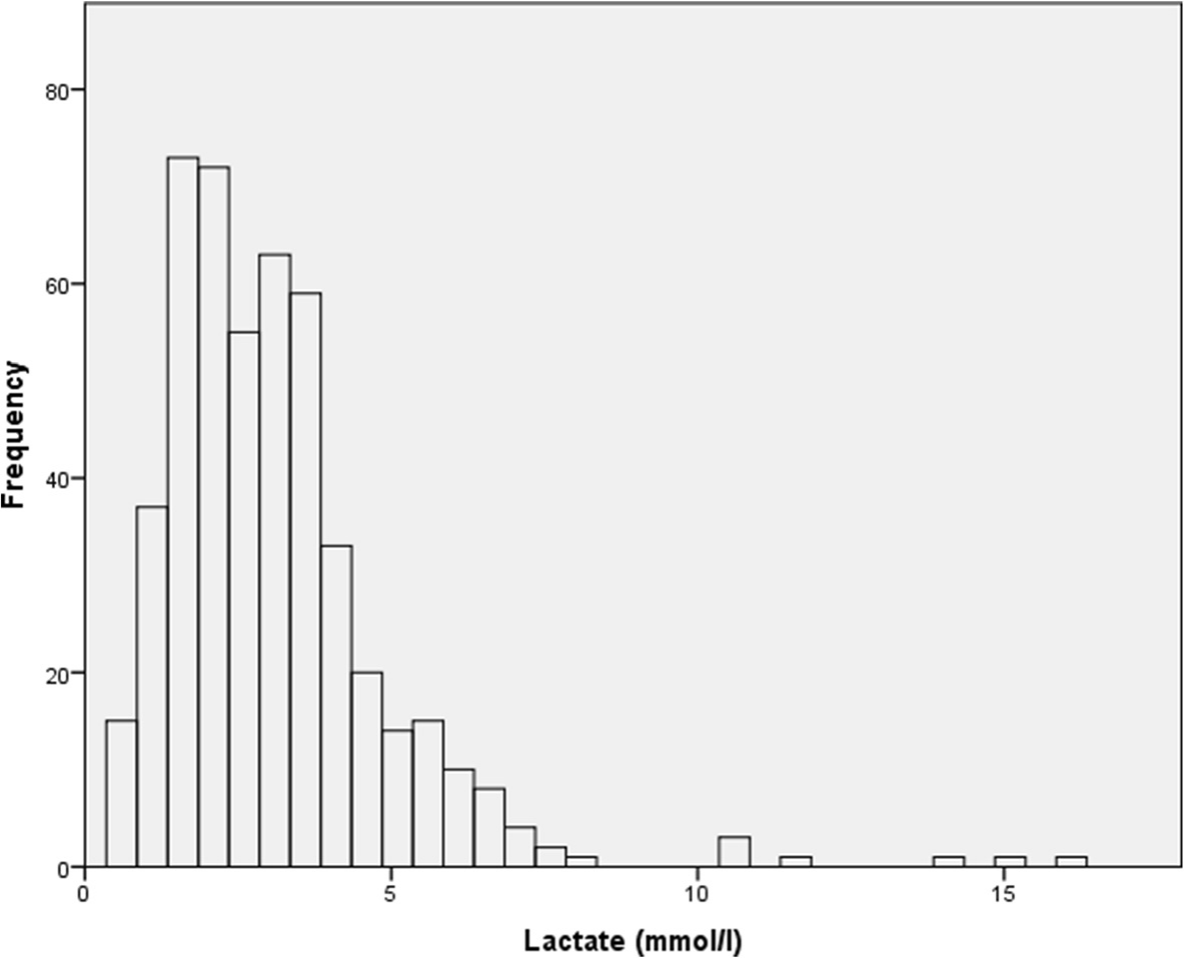
Distribution of arterial lactate concentration in 488 patients at the end of liver resection.

**Figure 2:**
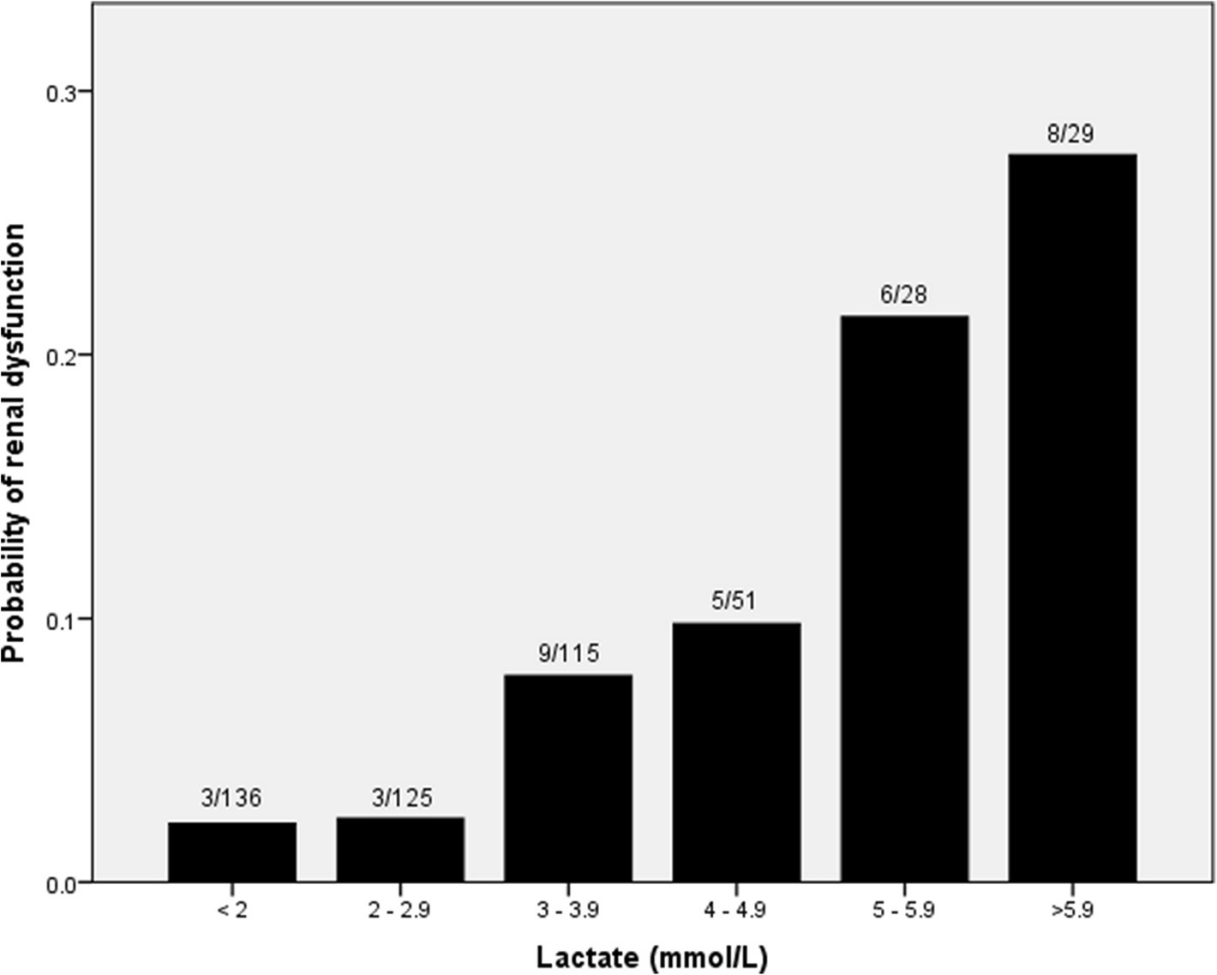
Probability of renal dysfunction after liver resection according to lactate concentration in 484 patients.

The median initial lactate concentration was 2.8 mmol/L (inter-quartile range = 1.9 to 3.9) and 350 patients (72%) had an elevated serum lactate concentration (≥2 mmol/L) (Figure 1). There was no difference in the lactate concentration taken prior to abdominal closure (n = 380, median 2.8 mmol/L, range 0.6 to 16.0) or immediately on arrival in the HDU (n = 108, median 2.8 mmol/L, range 0.6 to 14.0). The initial lactate concentration was noted to be associated with all recorded outcome measures (Table 4). Although major abnormalities of serum bilirubin and PT were rare in our series there was a weak correlation with initial lactate for both bilirubin (coefficient 0.41, *P* < 0.001) and PT (coefficient 0.37, *P* < 0.001), which was stronger for bilirubin. Similarly, there was a weak correlation with length of hospital stay (coefficient 0.28, *P* < 0.001). Of note the values for length of hospital stay include only survivors, and therefore exclude some patients who are likely to have high post-operative lactate levels. Renal dysfunction after liver resection was rare in this series (7.0%) but there was a correlation with lactate concentration (Table 4). Three of 137 patients (2.2%) with an initial lactate concentration less than 2 mmol/L who had creatinine measured developed renal dysfunction (negative predictive value (NPV) = 0.98) compared to 8 of 29 (27.5%) patients with an initial lactate greater than 6 mmol/L (positive predictive value (PPV) = 0.28) (*P* = < 0.001) (Figure 2). In 322 patients with a lactate concentration ≥2 and <6 mmol/L 23 developed renal dysfunction (7.1%).

**Table 4:** Univariate analysis of the association between lactate and postoperative outcomes for 488 patients undergoing liver resection

**n = 488**	**Co-efficient ± SD**	***P *** **value**
Peak bilirubin	0.146 ± 0.017	<0.001*
Peak prothrombin time	0.055 ± 0.002	<0.001*
Length of stay	0.046 ± 0.006	<0.001*
Renal dysfunction	0.324 ± 0.072	<0.001*
90-day mortality	0.373 ± 0.079	<0.001*

*Significant at level of *P* <0.05.

**Figure 3:**
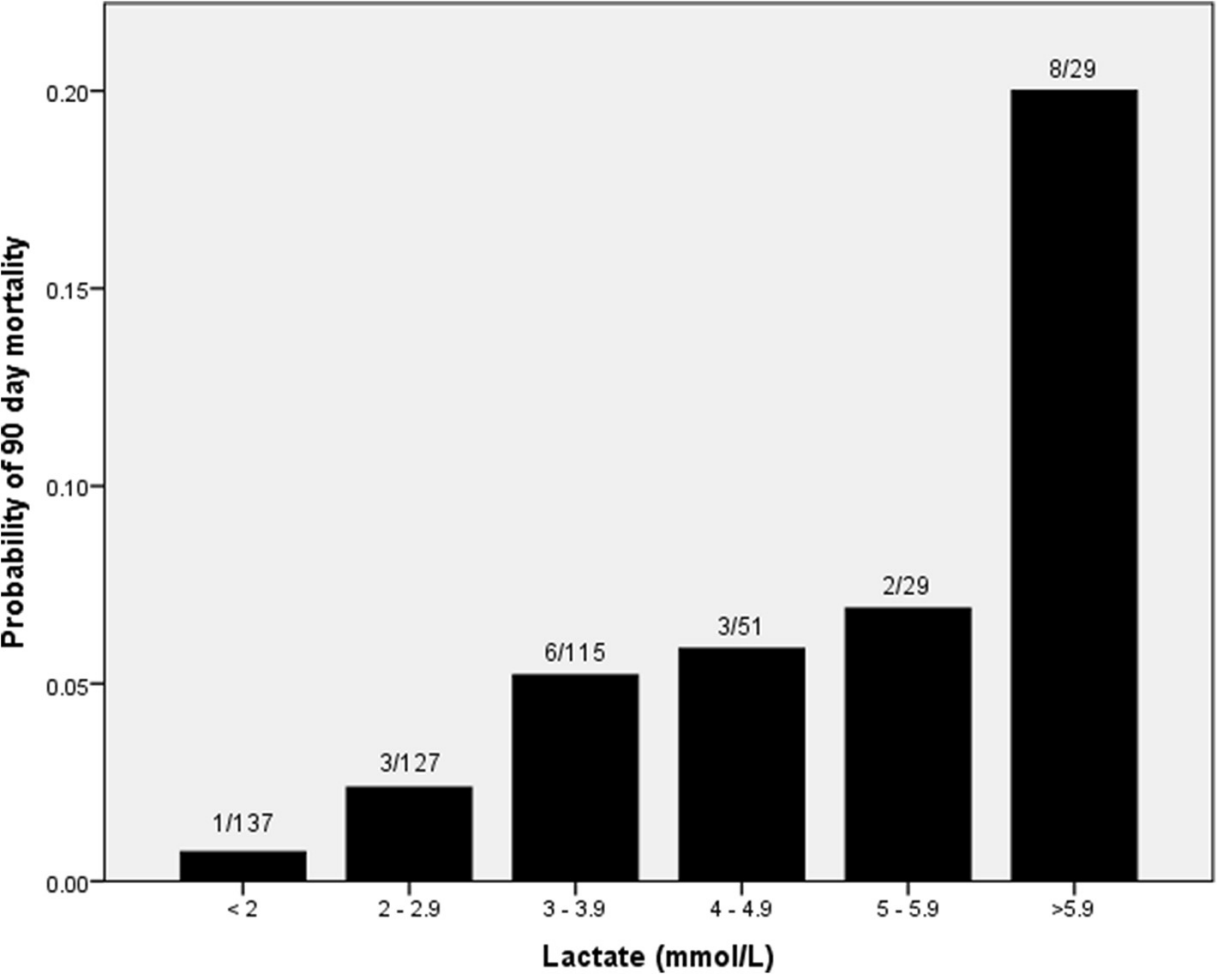
Probability of 90-day mortality after liver resection according to lactate concentration in 488 patients.

Similarly, there was a correlation between mortality in the 90-day period following liver resection and initial lactate concentration (Table 4). One of 138 patients (0.7%) with an initial lactate concentration <2 mmol/L died within this period, due to an anastomotic leak following colonic resection (NPV = 0.99), compared to eight of 29 patients with initial lactate ≥6 mmol/L (PPV = 0.28) (*P* = < 0.001) (Figure 3). The deaths in patients with lactate ≥6 mmol/L were due to liver failure in four patients, sepsis without liver failure in two patients, cardiac failure in one patient and ongoing malignancy in the other. Of the remaining 322 patients with lactate concentration ≥2 and <6 mmol/L there were 14 deaths within 90 days of surgery (4.3%).

Comparison of patients with initial lactate concentrations <2 mmol/L and ≥6 mmol/L revealed there were significantly more major resections performed (*P* < 0.001) and more patients with pre-operative diabetes (*P* < 0.001) in patients with a lactate concentration ≥6 mmol/L (Table 5). There was no significant difference in the use of pre-operative chemotherapy between these two groups (*P* = 0.351). The proportion of patients with both renal dysfunction and who died within 90 days was significantly higher in those with lactate concentrations ≥6 mmol/L (*P* < 0.001).

**Table 5:** Distribution of risk factors and outcomes in 138 patients with lactate <2 mmol/L and 29 patients with lactate ≥6 mmol/L undergoing liver resection

**Lactate**	**<2 mmol/L (n = 138)**	**≥6 mmol/L (n = 29)**	***P *** **value**
Major resection (%)	26 (18.8)	26 (89.7)	<0.001*
Pre-operative chemotherapy (%)	38 (27.5)	5 (17.2)	0.351
Pre-operative diabetes (%)	6 (4.3)	8 (27.6)	<0.001*
Post-operative renal dysfunction (%)	3 (2.2)	8 (27.6)	<0.001*
90-day mortality (%)	1 (0.7)	8 (27.6)	<0.001*

*Significant at level of *P* <0.05.

Regression analysis revealed that a pre-operative diagnosis of diabetes mellitus, the number of liver segments resected, the operating surgeon’s assessment of the health of the liver parenchyma, the operative blood loss and number of units of red cells transfused were all independently associated with initial lactate concentration at closure (Table 6). The only pre-operative factor associated with the post-operative lactate concentration was the presence of diabetes. On average, this increased the post-operative lactate concentration at any level by 20% compared to non-diabetics.

**Table 6:** Univariate and multivariate analysis of pre- and intra-operative factors associated with serum lactate concentration following liver resection in 488 patients

**N = 488**		**Univariate analysis**	**Multivariate analysis**	
**Factor**		***P *** **value**	**Co-ef +/− SD**	***P *** **value**
Age		0.246*		0.925
Gender		0.012*		0.129
Pathology	Benign vs. Primary	0.442		0.144
	Primary vs. Secondary	0.226*		0.878
Liver-directed chemotherapy		0.129*		0.219
Open or laparoscopic resection		0.009*		0.611
Radiofrequency ablation		0.191*		0.402
Wedge resection included		<0.001*		0.086
Bile duct reconstruction		0.004*		0.651
Number of segments resected		<0.001*	0.143 ± 0.012	<0.001†
Synchronous bowel procedure		0.516		
Surgeon’s assessment of liver		<0.001*	0.185 ± 0.042	<0.001†
Redo operation	1st vs. 2nd resection	0.268		
	2nd vs. 3rd resection	0.654		
Pre-operative diabetes		<0.001*	0.204 ± 0.064	0.002†
Body mass index		0.06*		0.905
ASA grade	1 vs. 2	0.014*		0.824
	2 vs. 3	0.709		0.872
P-POSSUM physiologic score		0.054*		0.221
Hepatic fibrosis/cirrhosis		0.667		
Pre-operative bilirubin		0.320		
Pre-operative haemoglobin		0.633		
Neutrophil:lymphocyte ratio		0.400		
Pre-operative albumin		0.399		
Pre-operative alkaline phosphatase		0.014*		0.775
Pre-operative creatinine		0.392		
Pre-operative glomerular filtration rate (GFR) >90 ml/min		0.042*		0.054
Blood loss (ml)	<500 vs. 500-999	<0.001*	0.131 ± 0.038	0.013†
	500-999 vs. >1000	0.435		0.884
Units of red cells transfused		<0.001*	0.043 ± 0.011	<0.001†

*Significant at the level of 0.25 for univariate analysis and included in multivariate analysis; †significant at the level of 0.05 for multivariate analysis.

## Discussion

The principal findings of this study are that higher initial serum lactate concentration after liver resection is associated with an increased risk of mortality and renal and liver dysfunction. Both the 90-day mortality rate and the rate of renal dysfunction in patients with initial lactate concentrations greater than 6 mmol/L were 28% compared to those patients with initial lactate concentrations less than 2 mmol/L where they were 0.7% and 2.2% respectively. Similarly, higher lactate concentration was associated with higher post-operative peaks in serum bilirubin concentration and PT, as well longer lengths of hospital stay.

These findings support and extend those of an earlier study [40] by demonstrating the association of post-operative lactate with renal and hepatic dysfunction and length of hospital stay in addition to mortality. Pre-operative diabetes mellitus, the surgeon’s assessment of the liver at laparotomy, the extent of liver resection, blood loss and the number of units of blood transfused are also shown to be associated with post-operative serum lactate concentration.

During cellular hypoxia pyruvate is diverted from the citric acid cycle and converted to lactate, reducing the amount of adenosine triphosphate (ATP) generated. This occurs in all metabolically active tissues including muscle, gut, liver, brain, erythrocytes and skin [41-43] and is exacerbated by intra-operative stresses including blood loss [42], endogenous release of stress hormones [44] and administration of pressor agents [45]. Liver ischaemia induced by handling of the liver during surgery and temporary inflow occlusion has been shown to lead to a rise in lactate [46]. Serum lactate can also be increased by transfusion of stored blood, which contains a higher concentration of lactate than fresh blood depending on length of storage [47]. Administration of Hartmann’s solution has been shown to have a small effect on serum lactate concentration [48]. A potential weakness of this study is that details of pressor agents were not recorded, which could affect the lactate concentration. Similarly precise details regarding intravenous fluid type and volume of fluid (colloid and crystalloid) were not recorded.

In addition to being a potential source of lactate the liver is the principle location of lactate metabolism, where it is converted back to glycogen, accounting for 70% of whole body lactate clearance [42]. No change in lactate metabolism has been demonstrated following recovery from partial hepatectomy in either rats [49] or humans [25], implying that the liver has a large functional reserve under physiological conditions of lactate production. However, the effects of intra-operative stress on hepatic glucose homeostasis have not been assessed, particularly when in combination with an extended hepatectomy. It is possible that inflow occlusion during resection and intra-operative handling of the liver lead to a temporary impairment of the ability of the liver to metabolise lactate. The finding of an association between the number of liver segments resected and the initial post-operative lactate supports this hypothesis. Diabetes is also known to be associated with impaired lactate metabolism via gluconeogenesis [42] and may account for the strong association with post-operative lactate in this series. Furthermore, the use of metformin in non-insulin-dependent diabetes has also been shown to increase lactate concentration [50]. The rise in serum lactate at the end of liver resection therefore may be due to a failure of lactate metabolism in addition to increased production during surgery.

Significantly, the use of pre-operative chemotherapy was not shown to be associated with elevation of post-operative lactate. This may be due to a policy of allowing a period of recovery after completion of pre-operative chemotherapy before undertaking surgery. Interestingly, the operating surgeon’s assessment of the liver parenchyma was associated with the post-operative lactate concentration. This finding suggests that patient co-morbidity was a more common cause of abnormal liver parenchyma than the use of liver-directed chemotherapy.

An important observation of this study is the relative rarity of major hepatic dysfunction following liver resection in this series with only one patient fulfilling the ‘50-50’ criteria [2], who subsequently recovered. Despite the infrequency of major disturbances of post-operative bilirubin and PT, there was an independent association with increasing concentration of post-operative lactate, demonstrating that even a minor degree of liver injury can lead to impaired lactate clearance or increase its production.

Renal dysfunction was also rare in this series, affecting 34 patients (7%) compared to 15% in a similar series [51]. The risk factors for post-operative renal dysfunction are likely to be similar to those in other forms of abdominal surgery, including blood loss and sepsis, which are also initiating factors for anaerobic metabolism and lactate production. This supports the value of initial lactate as an early predictor of renal dysfunction. Of note, the risk of renal dysfunction appeared to rise more rapidly when the post-operative lactate rose above 5 mmol/L (Figure 2). This suggests that the kidneys are able to tolerate a degree of oxidative stress to a threshold level beyond which the risk of damage rises rapidly.

There was a weak association between initial lactate concentration and length of hospital stay in the study (Table 4). However, this may also be affected by other factors such as post-operative complications, particularly bile leaks, and degree of social support.

The strongest association demonstrated was between lactate concentration and the risk of mortality. In a similar manner to renal dysfunction, there seems to be a threshold level of post-operative lactate of approximately 6 mmol/l above which the risk of 90-day mortality rises rapidly (Figure 3). Organ dysfunction was a major contributor to mortality in the series and initial lactate concentration is a valuable global marker of poor organ function in the early post-operative period, including cardiovascular, renal and hepatic dysfunction.

## Conclusions

These findings are of value in clinical practice as it may be possible to use the initial post-operative lactate concentration to determine the patient pathway in the early post-operative period. Patients with an initial post-operative lactate of less than 2 mmol/L have low rates of mortality and organ dysfunction and we are currently evaluating this criterion as a determinant of the need for post-operative critical care. In addition the correlation of post-operative lactate with subsequent organ dysfunction and mortality may allow its use as a single measure of the impact of innovations in operative technique or peri-operative care.

## Abbreviations

ASA: American society of anesthesiologists; ATP: Adenosine triphosphate; CUSA: Cavitron ultrasonic surgical aspirator; CVP: Central venous pressure; HDU: High dependency unit; LOS: Length of stay; NLR: Neutrophil to lymphocyte ratio; NPV: Negative predictive value; PHLF: Post-hepatectomy liver failure; P-POSSUM: Portsmouth physiologic and operative severity score for the enUmeration of mortality and morbidity; PPV: Predictive value; PT: Prothrombin time; REC: Research ethics committee; RIFLE: Risk, injury, failure, loss, and end-stage kidney disease.

## Competing interests

The authors declare that they have no competing interests.

## Authors’ contributions

MW and TS designed the study, collected data, analysed the data and drafted the manuscript. GS participated in the design of the study and performed the statistical analysis. TW, DB, PE, IA, SA and MB participated in the design of the study, collected data and drafted the manuscript. DS conceived the study, supervised its design and coordination and helped to draft the manuscript. All authors read and approved the final manuscript.

## Authors’ information

M.G. Wiggans and T. Starkie: joint first authors.
